# Pathomechanisms in central serous chorioretinopathy: A recent update

**DOI:** 10.1186/s40942-023-00443-2

**Published:** 2023-01-20

**Authors:** Arman Zarnegar, Joshua Ong, Tejaswini Matsyaraja, Supriya Arora, Jay Chhablani

**Affiliations:** 1 Department of Ophthalmology, University of Pittsburgh School of Medicine, Pittsburgh, PA USA; 2 Sarojini Devi Eye Hospital, Hyderabad, India; 3 Bahamas Vision Centre and Princess Margaret Hospital, Nassau, NP Bahamas

**Keywords:** Choroid, Central serous chorioretinopathy, Pachychoroid disease, Retina

## Abstract

**Background:**

Central serous chorioretinopathy (CSCR) is a potentially blinding choroidal disease. Despite decades of research, the pathological mechanisms of CSCR are still poorly understood. In recent years, there has been a strong emphasis on choroidal dysfunction as a primary cause of CSCR.

**Main Body:**

The concept of the pachychoroid disease spectrum and pachychoroid-driven processes are central to current theories regarding the pathophysiological underpinnings of CSCR. Choroidal hyperpermeability and subsequent leakage of fluid seen in CSCR may be due to several causes. Among them are venous congestion, inflammation, mineralocorticoid receptor activation, systemic factors including hemodynamic changes, obstructive sleep apnea, phosphodiesterase inhibitor use, pregnancy, and genetic predispositions. Congestion of vortex veins that drain blood from the choroid may contribute to the dilation of Haller vessels and cause fluid leakage. Vortex veins exit the eye through the sclera; thus, increased scleral thickness has been proposed to be a factor in venous congestion. Asymmetric vortex vein drainage may similarly result in congestion of the local venous system. Vortex vein anastomoses may overload the venous system and form secondary to venous congestion. Recent studies suggest inflammation and mineralocorticoid activation may factor into the development of CSCR, though more research in these areas is called for. Systemic conditions and genetics may predispose individuals to develop CSCR.

**Conclusions:**

By striving to understand the molecular and physiological mechanisms of this disease, we can better diagnose and treat CSCR to improve outcomes for patients.

## Background

Central serous chorioretinopathy (CSCR) is a vision-threatening disease of the pachychoroid disease spectrum that generally presents with central vision loss, metamorphopsia, central scotoma, and other visual disturbances. CSCR is characterized on imaging by the accumulation of subretinal fluid (SRF) in the macular region, which causes neurosensory retinal detachment [1]. On a demographic level, CSCR is more common in men, especially those around 40 years old [2]. A population-based study conducted by Kitzmann et al. reported an incidence of 9.9 cases per 100,000 men and 1.7 cases per 100,000 women in Olmsted County, MN, USA [2]. While revisions to the CSCR classification system have been proposed, the current standard is to describe CSCR as either acute or chronic depending on the duration of symptoms and persistence of SRF [3, 4]. The acute form often resolves spontaneously in most cases; however, if SRF persists for over three months, the disease is labeled as “chronic” with worse implications on visual acuity outcomes. Risk factors for developing CSCR have been extensively studied [5] and include heritable mutations in complement factor H gene, [6] hypertension, [7] endogenous steroid production and exogenous systemic corticosteroid use, [5, 8], the use of phosphodiesterase inhibitors [9], and pregnancy [10]. Current treatments for CSCR include photodynamic therapy, subthreshold laser, anti-corticosteroids, adrenergic blockers [11–13].

Despite extensive research, the pathophysiological underpinnings of CSCR still need to be fully elucidated. CSCR is thought to be caused by choroidal congestion, hyperpermeability, and retinal pigment epithelium (RPE) dysfunction. Current work centers around the involvement of the choroid. The choroid is a subretinal structure containing vessels that perfuse the outer and middle retina layers. Disruption of normal choroidal function has been strongly implicated as a primary cause of CSCR. In 1967, Gass first proposed that choroidal hyperpermeability drives the development of CSCR by increasing the hydrostatic pressure within the choroidal tissues [14]. While alternate hypotheses have been explored, Gass’ proposal was reinforced by imaging studies utilizing indocyanine green angiography [14].

The latest theories, such as the pachychoroid-driven process, build upon Gass’ work. The pachychoroid disease spectrum is a relatively novel concept that includes clinical conditions such as CSCR, pachychoroid neovasculoapthy (PNV), and polypoidal choroidal vasculopathy (PCV). Pachychoroid diseases are defined by (1) pachychoroid or increased choroidal thickness, (2) pachyvessels or dilated choroidal veins of the Haller layer [15], and (3) thinning of the inner choroid [16]. The pachychoroid-driven process is a sequence of events initiated by choroidal vessel dilation that culminates in choriocapillaris ischemia-induced SRF formation. While early ischemia can be noted in patients with acute CSCR as evidenced by decreased vessel density of the superficial choroid, serum and aqueous humor levels of vascular endothelial growth factor of these patients are not elevated compared to those of controls [17, 18]. The evaluation of intravitreal anti-vascular endothelial growth factor injections in acute CSCR has reportedly not demonstrated long-term improvements in visual acuity outcomes or choroidal thickness [19]. This process is believed to be at the center of CSCR pathology.

**Fig. 1:**
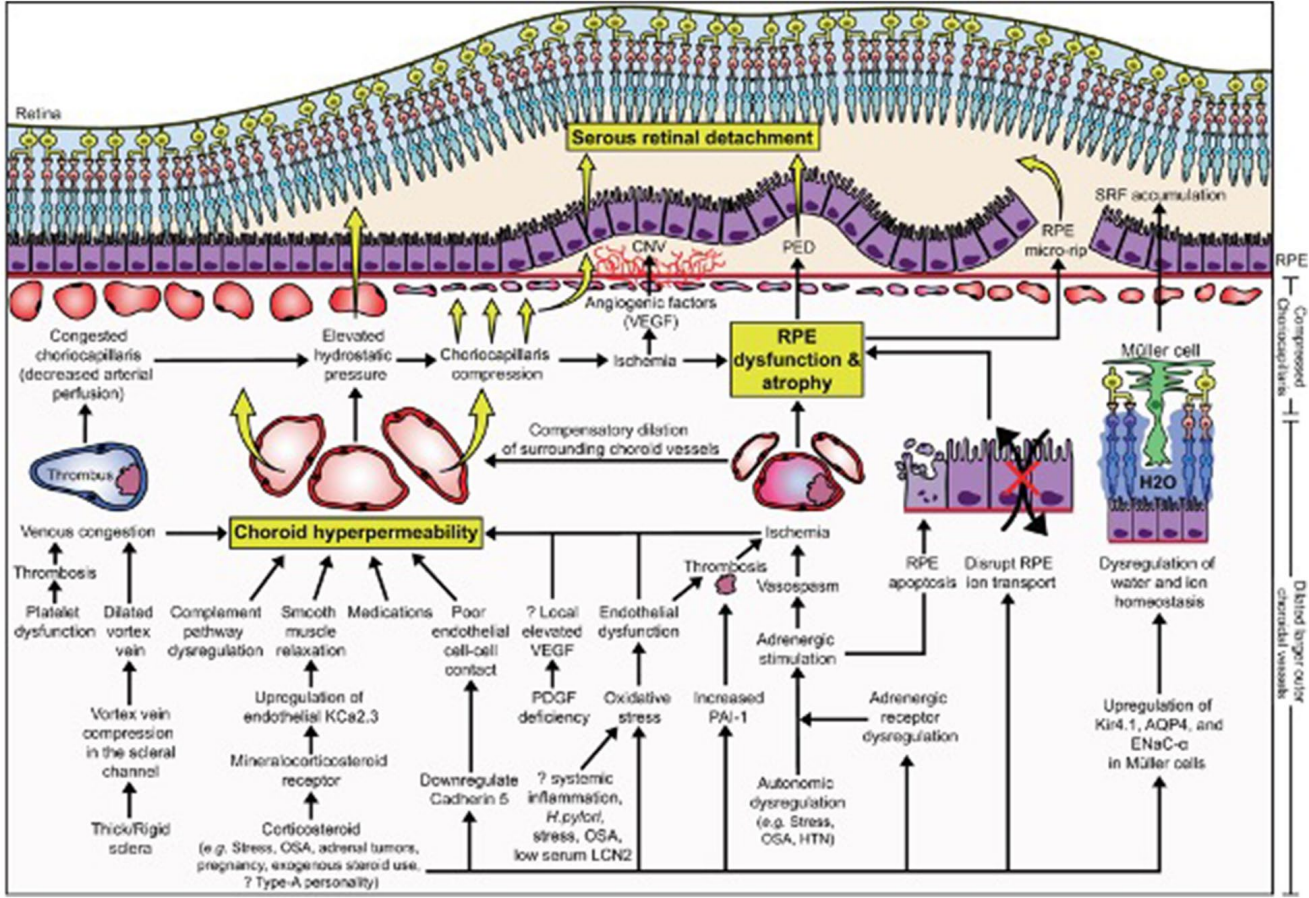
Molecular pathophysiological mechanisms causing serous retinal detachment, pigment epithelial detachment, and choroidal neovascularization in CSCR. The upstream mechanisms leading to choroid hyperpermeability include vortex vein compression, corticosteroid receptor activation, and oxidative stress/inflammation. Reprinted with permission from Kanda et al. [66] Pathophysiology of central serous chorioretinopathy: a literature review with quality assessment. Eye 36, 941–962 (2022) under Springer Nature and Copyright Clearance Center Rights Link License

Ultimately, there are many likely molecular and physiological factors that lead to choroidal hyperpermeability with subsequent serous retinal detachment. These pathophysiological factors include vortex venous congestion/compression, mineralocorticoid receptor activation, complement pathway dysregulation, inflammatory processes, and/or oxidative stress (Fig. 1). These factors that possibly increase the permeability of the choroidal vasculature will elevate hydrostatic pressure, which can directly cause serous retinal detachment and compression of the choriocapillaris. Compression of the choriocapillaris may cause ischemia leading to RPE atrophy and an increase of angiogenic factors such as vascular-endothelial growth factor. Loss of RPE barriers may lead to classic imaging findings in CSCR, including pigment epithelial detachment and SRF. The angiogenic component leads to choroidal neovascular membrane formation in CSCR. These downstream effects leading to CSCR findings may be due to a conglomerate of molecular and physiologic pathways. In this paper, we aim to review the recent advancements that contribute to our understanding of CSCR pathophysiology, namely venous congestion, inflammation, and mineralocorticoid receptor activation.

## Vortex vein congestion and central serous chorioretinopathy

**Fig. 2:**
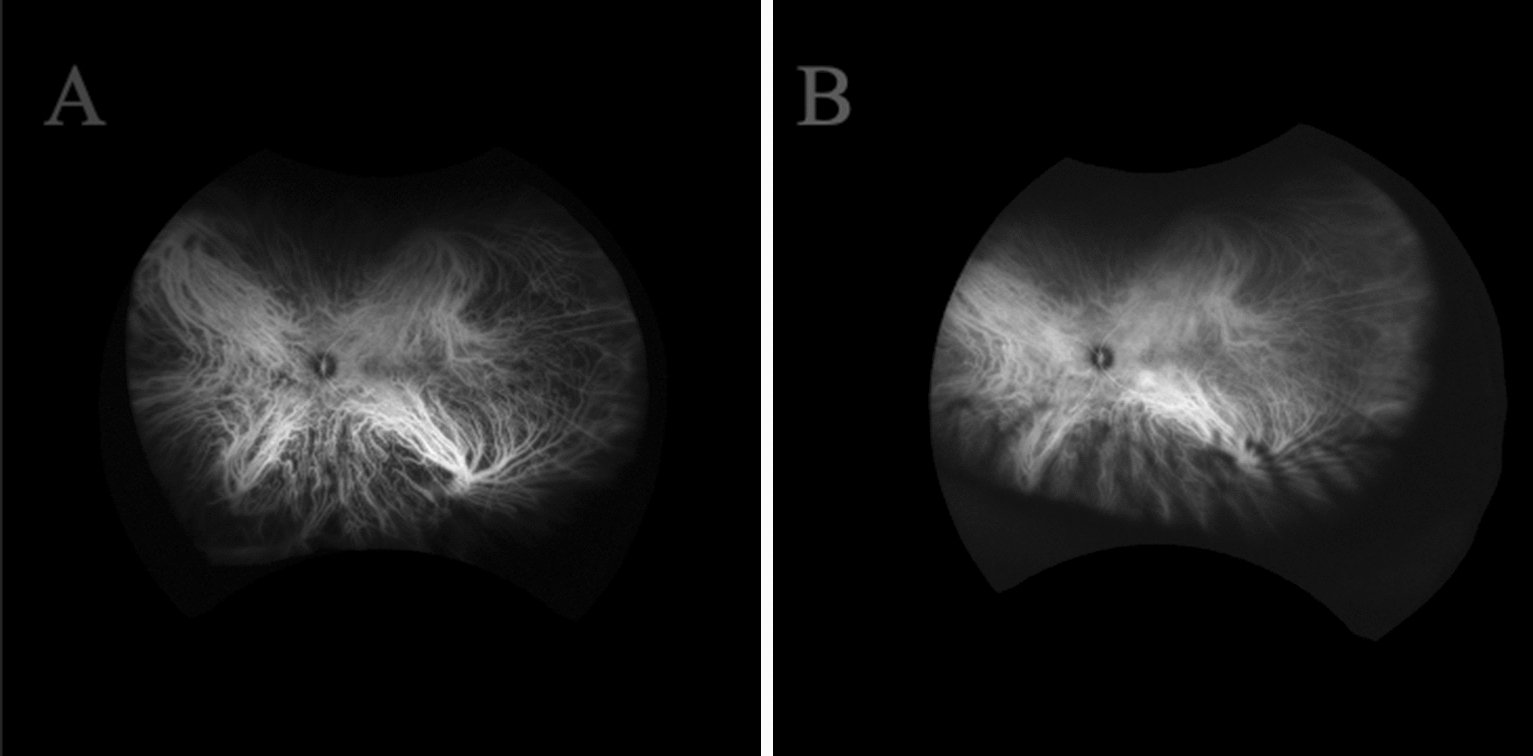
Indocyanine green angiography demonstrating vortex veins in the left eye of a patient with CSCR. (**A**) Early and (**B**) late phases show dilated vessels and late leakage

Venous congestion has been highlighted as a potential mechanism of pachyvessel and pachychoroid formation [20, 21]. In a brief review of relevant anatomy, blood perfusing the choroid arrives via short and long posterior ciliary arteries, which are branches of the ophthalmic artery. The choriocapillaris, the inner layer of the choroid, holds fenestrated capillaries that allow for the exchange of gases and nutrients [22]. Venous drainage from retinal structures involves vortex veins that drain four quadrants of choroidal vasculature and empty into the superior ophthalmic veins (Fig. 2). These systems exhibit a degree of anastomoses in healthy vs. diseased eyes. Obstruction of vortex veins, especially the dominant vortex vein, which drains the macula, may contribute to the engorgement of Haller vessels and the development of SRF.

The dilatation of vortex veins occurs at their distal ends, suggesting that the elevated retrograde venous pressure is transmitted to the beginning of vortex veins. Hence the dilatation is marked at the level of vortex veins and moderate at the Sattler layer without any dilatation involving the choriocapillaris. Pang et al. observed dilation of one or more vortex veins in 83.3% of CSCR eyes imaged with ultra-widefield indocyanine green angiography and proposed choroidal congestion in the vortex veins may be the cause of CSCR [23].

**Fig. 3:**
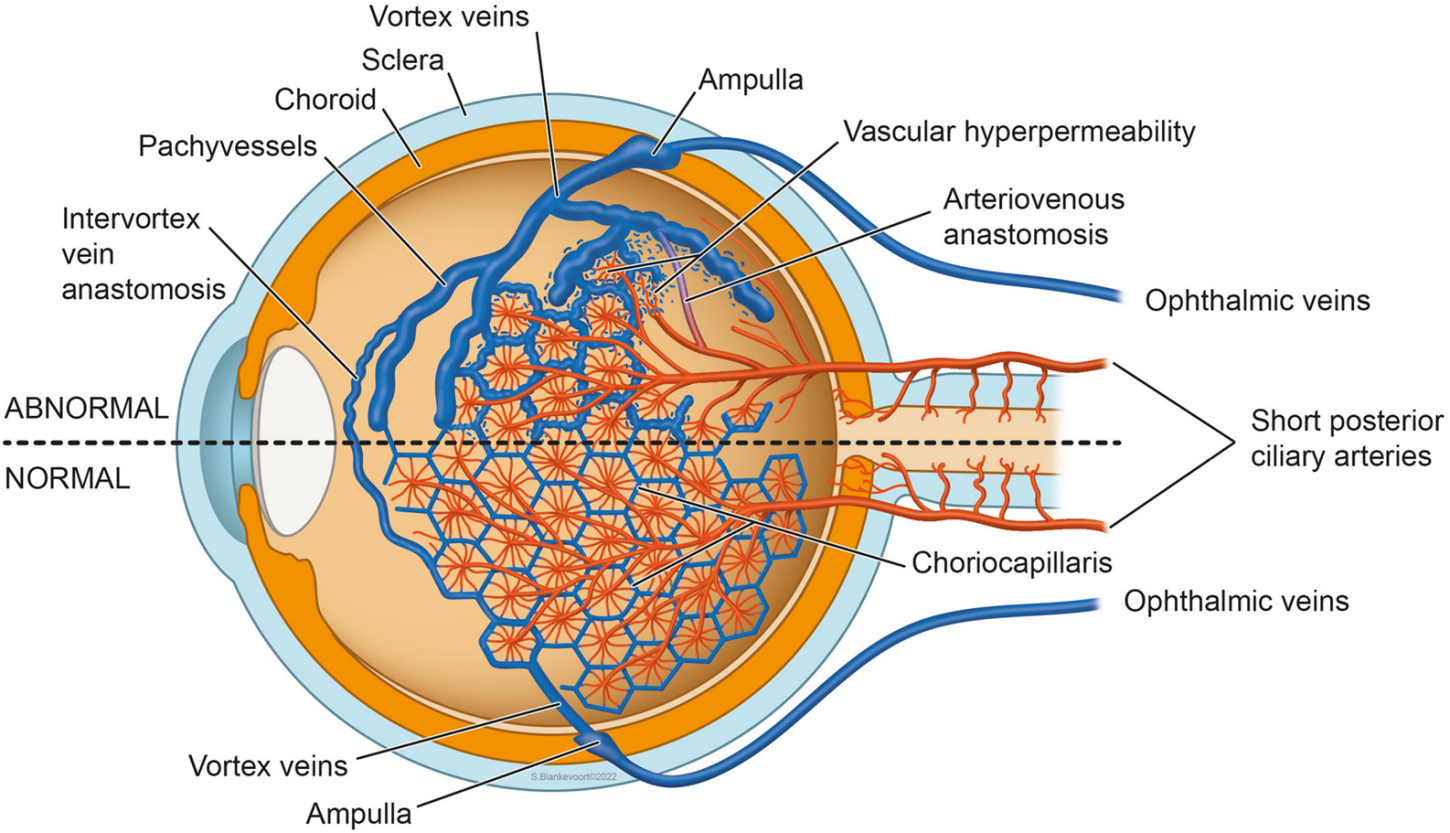
Diagram demonstrating choroidal arteriovenous vasculature. Healthy (bottom) and diseased (top) choroidal arteriovenous vasculature. In the abnormal choroidal arteriovenous vasculature, pathological anastomoses that bypass the capillary bed may form to cause an increased arterial filling in the vortex veins, leading to vortex vein congestion. This engorgement can lead to choriocapillaris congestion, leading to the release of angiogenic factors and the breakdown of the RPE as seen in CSCR. Reprinted with permission from Brinks et al. [24] Choroidal arteriovenous anastomoses: a hypothesis for the pathogenesis of central serous chorioretinopathy and other pachychoroid disease spectrum abnormalities. (2022) under Creative Commons Attribution-NonCommercial-NoDerivatives 4.0 International (CC BY-NC-ND 4.0) License (https://creativecommons.org/licenses/by-nc-nd/4.0/legalcode)

Brinks et al. [24] hypothesized that abnormal choroidal arteriovenous anastomoses may lead to CSCR progression. Anastomoses may bypass the choriocapillaris, leading to direct arterial filling of these veins, engorged vortex veins, and ultimately venous overload (Fig. 3). These anastomoses may lead to congestion of blood flow in the choriocapillaris, which may result in effects such as the increased release of angiogenic factors and breakdown of the RPE barrier. As such, the study of vortex vein hemodynamics has yielded novel concepts of CSCR pathogenesis.

### Scleral thickening

**Fig. 4:**
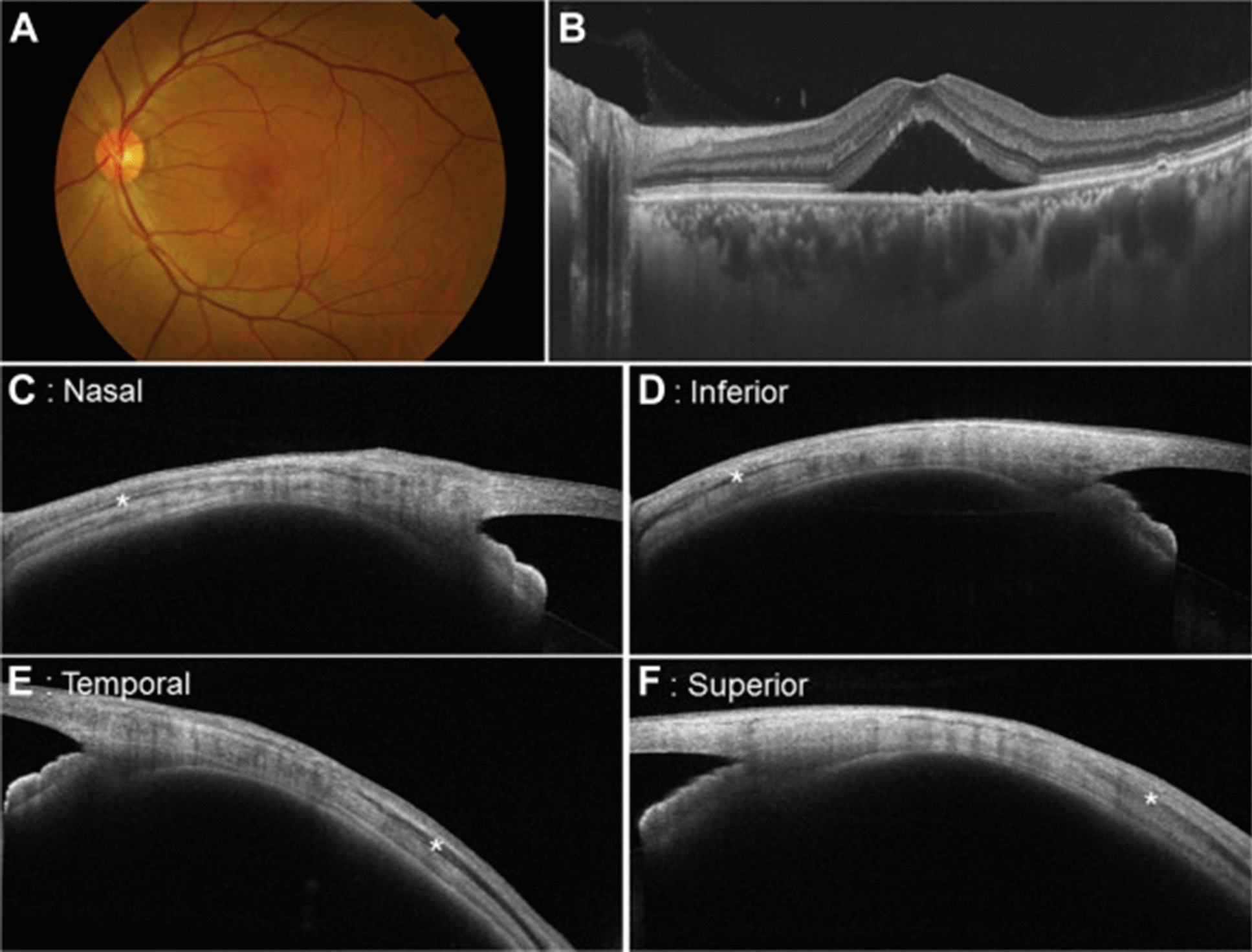
Swept source anterior segment OCT of the left eye of a 45-year-old male with CSCR. A color fundus photograph (**A**) demonstrates neurosensory retinal detachment secondary to SRF accumulation in the macula. Swept source OCT (**B**) shows SRF in the fovea with a subfoveal choroidal thickness of 469 μm and Haller vessel dilation. Scleral thickness was measured via anterior segment swept source OCT at nasal (**C**), inferior (**D**), temporal (**E**), and superior (**F**) regions with thicknesses of 455 μm, 485 μm, 442 μm, and 523 μm, respectively. Asterisks represent rectus muscles. Reprinted with permission from Imanaga et al. [26], under Creative Commons Attribution-NonCommercial-NoDerivatives 4.0 International (CC BY-NC-ND 4.0) License (https://creativecommons.org/licenses/by-nc-nd/4.0/legalcode)

Scleral thickening may mechanically obstruct choroidal venous outflow. Vortex veins drain through the sclera, penetrating the tissue for 4 mm. Increased resistance to venous flow may thus be conferred by the sclera in scleral thickening, for example, in hyperopic eyes [25]. Imaging studies have recently employed swept source optical coherence tomography (OCT) of the anterior segment to determine scleral thickness. Imanaga et al. [26] demonstrated increased anterior scleral thickness under the rectus muscle in eyes with CSCR compared to healthy controls in the first study using swept source anterior segment OCT (Fig. 4). Lee et al. [27] and Fernández-Vigo et al. [28] conducted similar studies using swept source OCT that showed increased anterior scleral thickness in CSCR compared to control measured under the lateral rectus and 0 and 2 mm from the scleral spur, respectively.

No OCT platform is currently available for accurately and directly measuring scleral thickness at the posterior pole, though. The utility of these anterior segment imaging methods is, therefore, somewhat limited because posterior scleral thickness must be inferred from anterior scleral thickness measurements. Spaide et al. [29] used contact B-scan ultrasonography probes with a 20 MHz annular phased array transducer to examine equatorial and posterior scleral thicknesses in 79 eyes with CSCR. Equatorial and posterior scleral thicknesses were each significantly greater in the CSCR group compared to the control group.

### Asymmetric vortex veins

**Fig. 5:**
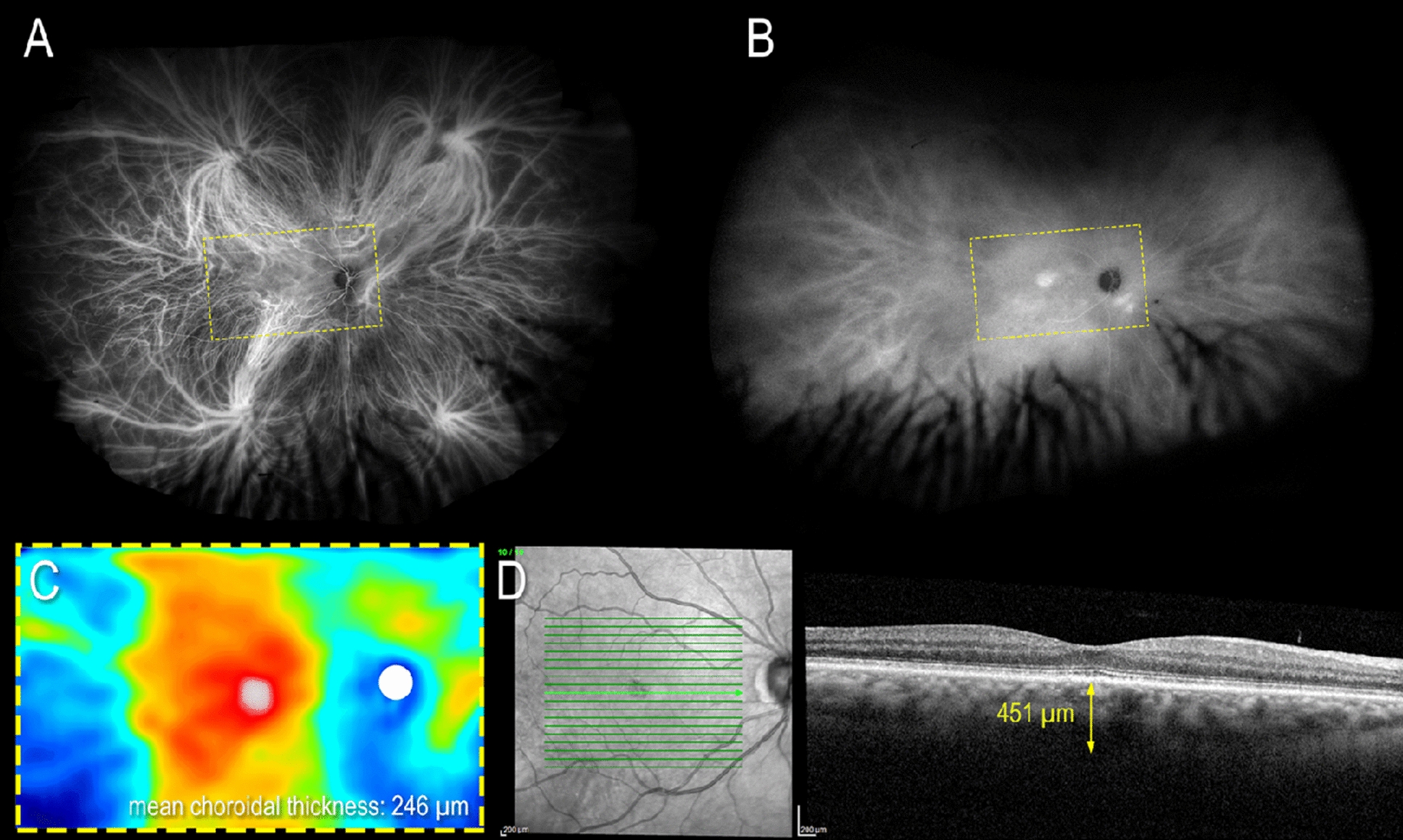
Multimodal imaging of the right eye of a 62-year-old woman with pachychoroid pigment epitheliopathy. (**A**) Early to mid-phase ultra-widefield indocyanine green angiography exhibits asymmetric drainage within the vortex venous system. Choroidal hyperpermeability can be observed in the macula and inferonasal peripapillary areas in a late-phase ultra-widefield indocyanine green angiography (**B**). Choroidal thickness maps in (**C**) are taken from the region marked by yellow dashed boxes in (**A, B**). Regions of choroidal hyperpermeability from (**B**) also exhibit increased choroidal thickness in (**C**) on swept-source OCT imaging. Spectral-domain OCT of the fovea shows a nonvascularized shallowed irregular pigment epithelial detachment (**D**). Reprinted with permission from Bacci et al. [30], under Creative Commons Attribution-NonCommercial-NoDerivatives 4.0 International (CC BY-NC-ND 4.0) License (https://creativecommons.org/licenses/by-nc-nd/4.0/legalcode)

A considerable proportion of healthy eyes exhibit asymmetric venous outflow pathways, which may predispose individuals to pachychoroid diseases [30–32]. A 2018 study by Hiroe and Kishi demonstrated that of 35 eyes with CSCR imaged with swept source OCT, all 35 possessed asymmetry in venous drainage compared to 38% of healthy eyes [32]. Bacci et al. [30] conducted a retrospective analysis of ultra-widefield indocyanine green angiography in 52 eyes with pachychoroid diseases, predominantly including eyes with simple and complex CSCR. They identified increased drainage through nasal vortex veins in diseased eyes compared with control eyes. Bacci’s group proposed that asymmetric vortex veins may cause venous overload, especially in the region of the macula (Fig. 5). Terao et al. [25] found that eyes with CSCR and vortex vein asymmetry had shorter mean axial lengths and greater subfoveal choroidal thicknesses than eyes with CSCR and no asymmetry.

In eyes with symmetric venous drainage, congestion of a unilateral vortex vein can be compensated for by contralateral venous drainage. However, in eyes with asymmetric venous drainage, congested blood flow in the dominant vein has no alternative drainage route.

The choriocapillaris has a sinusoidal structure with a wide lumen that allows 20-fold faster choroidal blood flow than the retina. Because the choroid has no tissue capillary complex between the arterioles and venules, the choriocapillaris receives direct pressure from both sides via the branches of the short posterior ciliary arteries and collecting venules of the congested vortex vein. The choriocapillaris has no tissue blood barrier like the retinal capillaries but has fenestrations in its inner wall. Elevated venous pressure might enhance the passage of water and small molecules through the fenestration.

### Vortex venous anastomoses

**Fig. 6:**
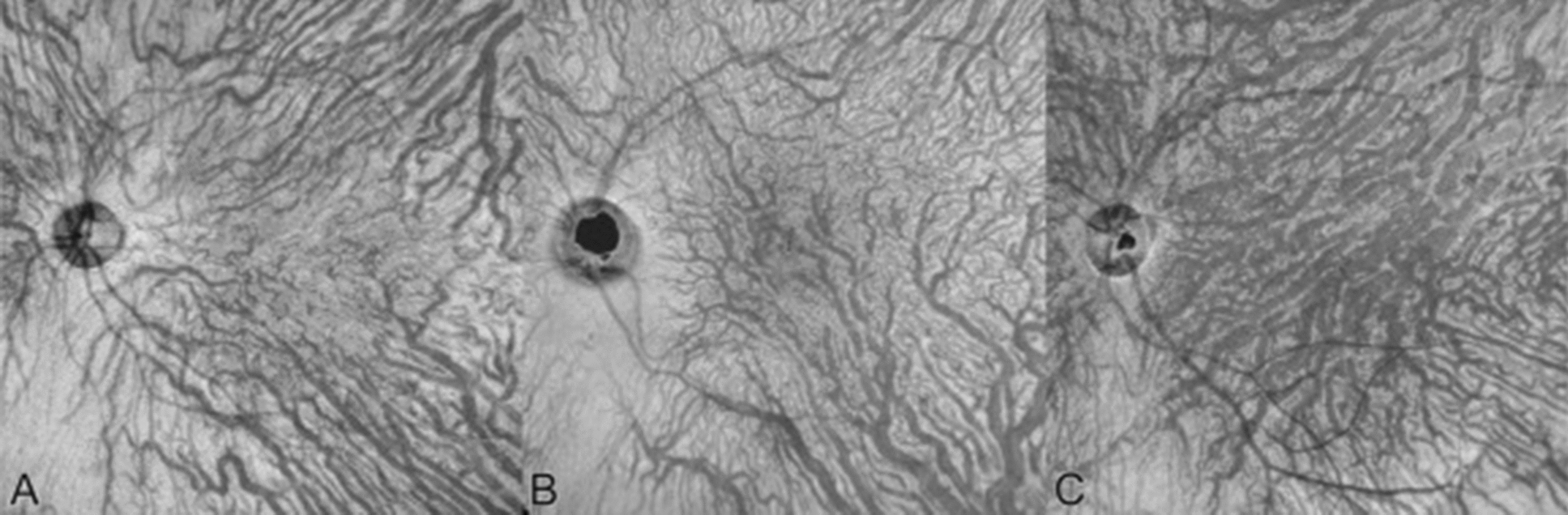
En face OCT images of the choroid. (**A**) Symmetrical venous drainage involving the superotemporal and inferotemporal vortex veins in a healthy eye. (**B, C**) Vortex vein anastomoses secondary to venous congestion in an eye with CSCR. Reprinted with permission from Spaide et al. [21], under Creative Commons Attribution-NonCommercial-NoDerivatives 4.0 International (CC BY-NC-ND 4.0) License (https://creativecommons.org/licenses/by-nc-nd/4.0/legalcode)

A potential consequence of increased venous hydrostatic pressure due to congestion is the development of venous anastomoses within the vortex vein system (Fig. 6). Matsumoto et al. [33] characterized the degree of superior and inferior vortex venous anastomoses of pachychoroid diseases, including CSCR, using en face OCT images. 90.2% of eyes with CSCR demonstrated anastomoses compared to 40.0% of healthy control eyes. Furthermore, 95.1% of eyes with PNV and 100% of eyes with PCV had anastomoses compared to their healthy controls (48.0% and 36.0%, respectively). Matsumoto and colleagues [34] later examined eyes with CSCR, PNV, and PCV to compare the vascular diameter of vortex veins across these diseases using binarized en face OCT. Central choroidal thickness and the vortex vein diameter was greatest in eyes with CSCR and decreased in eyes with PNV and PCV, but fewer eyes with CSCR demonstrated anastomosis between superior and inferior vortex veins than eyes with PNV and PCV. CSCR is thought to progress to PNV then PCV within the pachychoroid disease spectrum. Their group thus concluded that progressively increasing anastomoses across these diseases may alleviate venous congestion and reduce choroidal thickness.

## Inflammation and central serous chorioretinopathy

The effect of inflammation on the choroid in CSCR pathogenesis has yet to be completely understood, particularly because of the apparent relationship between endogenous or exogenous steroid exposure and the development of CSCR. Because steroids aggravate rather than improve CSCR, the proposed mechanism of inflammation causing CSCR could be considered steroid-insensitive inflammation. While some have identified elevated proinflammatory cytokines, such as IL-6, in CSCR patients, a link between CSCR and systemic inflammation has yet to be definitively established [35]. Nonetheless, the fact that choroidal hyperpermeability and leakage are seen in CSCR has led researchers to further investigate inflammatory markers in this process [7]. Recently, studies have examined serum markers of inflammation as predictors of CSCR development or severity. Sirakaya et al. examined monocyte-to-high-density lipoprotein ratios, given their relevance in disease states such as diabetic retinopathy [36, 37]. Monocytes are recruited in inflammatory states, while high-density lipoproteins counteract inflammatory processes. Their group identified a statistically significant increase in monocyte-to-high-density lipoprotein ratio in patients with acute CSCR compared to healthy controls. Limon et al. [38] conducted a study evaluating serum fibrinogen/albumin ratios in patients with acute and chronic CSCR. Results showed that fibrinogen/albumin ratios were significantly elevated in patients with acute CSCR compared to those with chronic CSCR or healthy controls. A study by Matet et al. [39] reported decreased serum lipocalin 2, an acute-phase reactant with both anti- and pro-inflammatory effects, in patients with acute and chronic CSCR relative to healthy controls.

Conversely, Zola et al. [40] studied serum levels of galectin 3, a proinflammatory marker, and reported a significant decrease in galectin 3 levels in patients with acute CSCR compared to healthy controls. In a 2019 study by Bahadorani et al., the role of topical non-steroidal anti-inflammatory agents in treating CSCR was assessed [41]. Despite the retrospective nature of the study and sample size of 27 patients, their group identified a significant decrease in both central macular thickness and SRF in eyes with CSCR that received topical non-steroidal anti-inflammatory agents compared to those who did not.

## Mineralocorticoid agonism and central serous chorioretinopathy

**Fig. 7:**
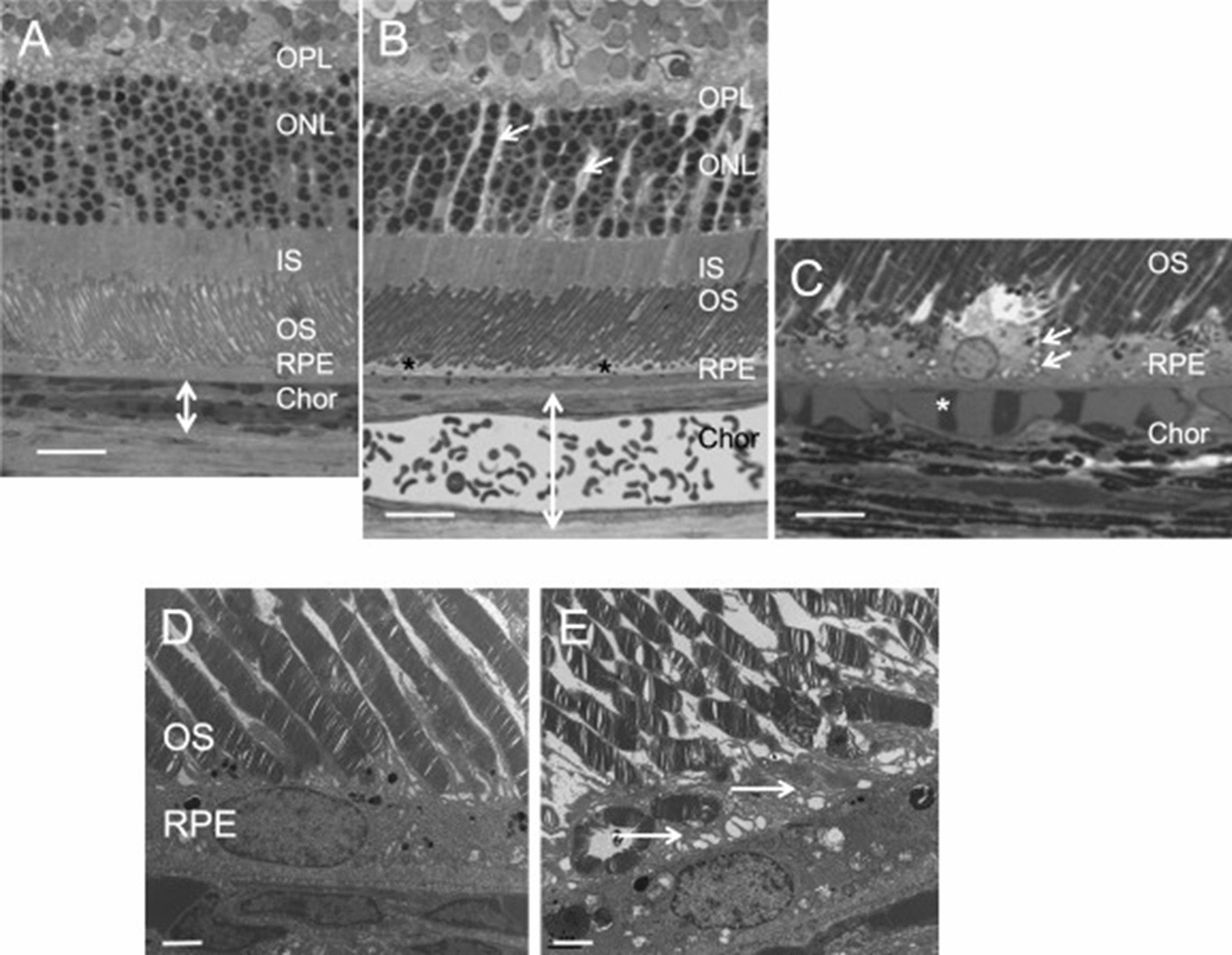
Effects of aldosterone injection in rat eyes. Control rat eyes (**A, D**) and aldosterone-injected rat eyes (**B, C, E**). Intravitreal injection of aldosterone in rat eyes in semi-thin sections shows (**B**) swollen Müller cells (arrows), thickened retinal pigment epithelial apical microvilli (stars), and thickened choroid (bidirectional arrows). Additionally, engorged choroidal vessels can be appreciated (**C**, star). Transmission electron microscopy (**E**) also shows an increased thickness of retinal pigment epithelial apical microvilli (arrows). INL, inner nuclear layer; IS/OS, inner and outer segments of photoreceptors. Bar: A and B, 20 μm; C, 10 μm; D and E, 2 μm. Reprinted with permission from Daruich et al. [5], under Creative Commons Attribution-NonCommercial-NoDerivatives 4.0 International (CC BY-NC-ND 4.0) License (https://creativecommons.org/licenses/by-nc-nd/4.0/legalcode)

**Fig. 8:**
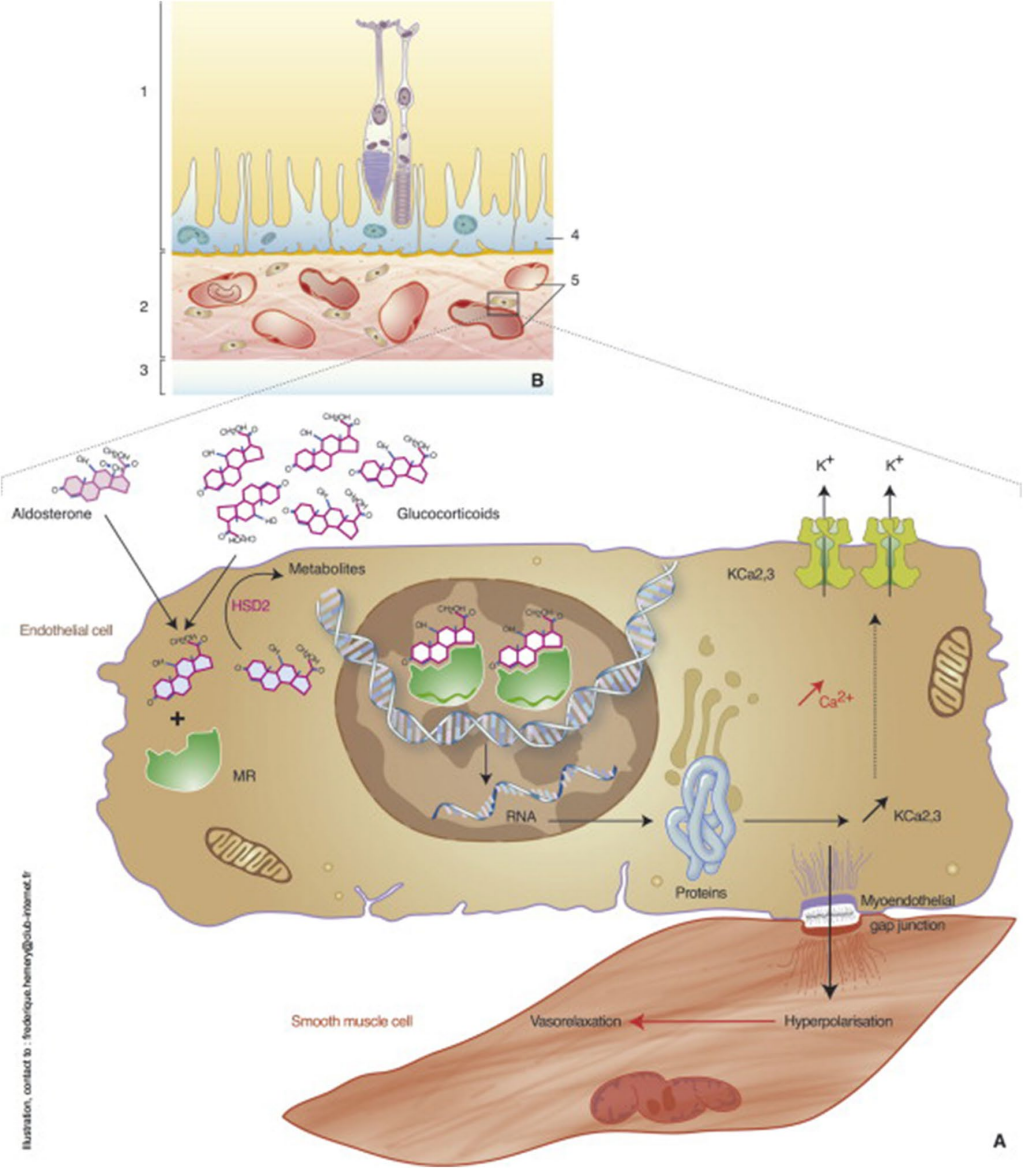
Mechanisms of choroidal vascular dilation via activation of mineralocorticoid receptors. Choroidal vascular endothelial cells express mineralocorticoid receptors (MR) which can be activated by aldosterone and glucocorticoids with the downstream effect of inducing vasodilation. Reprinted with permission from Daruich et al. [5], under Creative Commons Attribution-NonCommercial-NoDerivatives 4.0 International (CC BY-NC-ND 4.0) License (https://creativecommons.org/licenses/by-nc-nd/4.0/legalcode)

Studies have linked systemic corticosteroid use and elevated endogenous cortisol levels to CSCR [42, 43]. Based on experiments in animal models, aldosterone bound and activated mineralocorticoid receptors, causing vasodilation of choroidal vessels (Figs. 7, 8) [44]. A curious phenomenon is the relative lack of CSCR development following intraocular corticosteroid injections. Zola et al. hypothesized that the hypothalamic–pituitary–adrenal axis is inhibited by intraocular corticosteroids [45]. To test their hypothesis, rats were injected with intraocular dexamethasone daily for five days, and levels of corticosterone in ocular media were measured on day five. Corticosterone was elevated at endpoint, suggesting hypothalamic–pituitary–adrenal axis brake was achieved. Further, mineralocorticoid receptor/glucocorticoid receptor ratios had increased in choroidal tissues indicating mineralocorticoid overactivation.

Two studies recently examined choroids of patients with Cushing syndrome, or hypercortisolism. Wang et al. [46] identified increased choroidal thickness in patients with Cushing syndrome, and Eymard et al. [47] more commonly observed pachyvessels and pachychoroid diseases in patients with Cushing syndrome than in healthy controls. A current treatment for CSCR involves mineralocorticoid receptor antagonism, given the principles discussed in this section. In practice, however, these drugs have not provided significant improvements in visual acuity and SRF resolution [48]. The basic mechanism here is that aldosterone and cortisol bind to mineralocorticoid receptors and glucocorticoid receptors, respectively, but it is because of the structural similarity between these two receptors that lead to the cross-reactivity of glucocorticoids and mineralocorticoids leading to the hyperpermeability.

## Systemic associations of central serous chorioretinopathy

Several systemic associations have been reported in patients with CSCR, suggesting that CSCR could be a part of a spectrum of generalized vascular disorders rather than an isolated choroidal vasculopathy. Increased sympathetic activity with a significant sympathetic-parasympathetic imbalance can lead to modulation of the blood flow in the choroid. This increased sympathetic activity which forms the primary precursor of hypertension, explains the increased incidence of CSCR in patients with hypertension. Drastic changes in arterial blood pressure can injure arterial walls in the choroid, thus causing CSCR [49].

Studies using indocyanine green angiography of patients with CSCR have revealed hyperpermeability with evidence of ischemia and the congestion of choroidal lobules [50].

Being modifiable, stabilization of BP towards lower levels may also aid in reducing the risk of recurrence. In a study by Erol et al., based on the major capillary changes observed using Nailfold video capillaroscopy, they observed that digital microcirculation in patients with CSCR is affected by capillary changes such as extravasation and tortuosity, suggesting CSCR is a part of systemic micro vasculopathy [51].

Autoregulation of the choroidal vasculature in response to systemic hemodynamic fluctuations may be dysregulated in patients with CSCR. In instances of heightened cardiac output, such as exercise in healthy patients, the choroidal vessels in Sattler’s and Haller’s layers vasoconstrict to maintain constant blood flow [52]. In patients with CSCR, choriocapillaris blood flow increases compared to that of controls in states of increased perfusion, suggesting regulatory mechanisms are disrupted [53]. At baseline, individuals with CSCR were identified to have elevated systolic and diastolic blood pressure compared to healthy counterparts [53]. When performing physical exercise in this study, the patients with CSCR exhibited blood pressure readings consistent with hypertension. Vigorous physical activity was found to be associated with a greater risk of developing CSCR. The joint effect of heightened systolic and diastolic blood pressures and exercise in patients with CSCR is an increase blood flow through the choroid that cannot be countered adequately by autoregulatory mechanisms. Ultimately, this process may contribute to downstream vortex venous congestion as the veins drain through the sclera [54].

Alterations in choroidal blood flow and vascular density can be monitored carefully with OCT angiography, a non-invasive imaging modality. OCT angiography of choriocapillaris in patients with CSCR reveals areas of ischemia, stasis, and hypoperfusion surrounded by regions of greater blood flow [55]. Nicoló et al. conducted a study using swept-source OCT angiography to assess vascular flow area in eyes with CSCR. Their results showed reduced vascular flow area in the choriocapillaris, but increased blood flow in Sattler’s and Haller’s layers [56]. These vascular changes might contribute to SRF formation. Physical exercise may improve visualization of Type 1 choroidal neovascularization within flat-irregular pigment epithelial detachments as captured by OCT angiography [57].

Phosphodiesterase inhibitor use has been associated with CSCR onset in a number of studies. Roy et al. identified a case of a 45-year-old man without prior steroid use or Type A personality developing CSCR after initiation of tadalafil therapy [9]. A similar case was reported by Mohammadpour et al. [58] in a healthy, young male patient on sildenafil. Phosphodiesterase inhibitors increase choroidal blood flow via nitric oxide-mediated vasodilation and may thicken the choroid. Sushma et al. [59] described increased mean choroidal thickness in patients on sildenafil at one hour and three hours after ingestion. The reported ocular effects of phosphodiesterase inhibitors warrant its consideration as a precipitant of CSCR.

Studies have shown that patients with obstructive sleep apnea (OSA) also have increased sympathoadrenal activity, evidenced by increased levels of urinary metabolites of catecholamines in these patients. In addition, apneic events, both directly and indirectly, can also lead to increased cortisol levels by disrupting the hormone regulatory response and activating the hypothalamic–pituitary–adrenal axis. Thus, the increased stress hormones seen in both patients with CSCR and OSA may explain the association between the two conditions, suggesting that OSA may be an independent risk factor for developing CSCR. Wu et al. conducted a study to investigate the association of OSA with CSCR. They utilized enhanced depth imaging to analyze subfoveal choroidal thickness changes in this patient population. Their study confirmed that patients with CSCR tend to have OSA and that moderate to severe OSA can lead to smaller subfoveal choroidal thickness on enhanced depth imaging OCT [60].

CSCR has been associated with type A personality disorder characterized by ambitious, competitive, and aggressive nature, a disorder linked to excess catecholamine and cortisol levels. HPA axis changes are well known in CSCR patients, and cortisol levels were correlated with personality alterations [61]. More recently, studies have shown the possible association between narcissistic personality and the use of sympathomimetic drugs [5], which can be related to the altered reactivity profile of CSCR patients to sympathetic stimuli.

Despite the marked male predisposition in the incidence of CSCR, pregnancy is a recognized risk factor. The occurrence of CSCR in pregnancy may be because of the increased endogenous corticosteroid levels associated with pregnancy. In addition, the increased blood volume and other hemodynamic changes that occur during pregnancy may be implicated in the development of CSCR [62].

## Genetic associations of central serous chorioretinopathy

Several cases of familial CSCR have been reported implying a genetic association with CSCR. The most promising evidence comes from the observation by Weenink et al., in which 14 out of 27 families of CSCR patients (52%) had at least one relative with multiple areas of RPE atrophy or fundus lesions suggestive of chronic CSCR [63]. Although no clear pattern of inheritance has been established, a study by Lehmann et al. found that 50% of the eyes of relatives of CSCR patients from five families had a choroid thicker than 395 μm, suggesting that pachychoroid could be an inherited condition with a possible dominant transmission pattern [64].

The association of CSCR with CFH has been well reported, few studies showed the association of CSCR with Cadherin-5. CDH5 is localized to endothelial cell junctions, including those between endothelial cells in the human choroid. As described for other vascular beds, CDH5 protein was localized to choriocapillaris and larger vessels in human choroid.

The protein encoded by the CDH5 gene is a calcium-binding cell–cell adhesion glycoprotein. The protein plays an important role in endothelial cell biology by controlling the cohesion and organization of the intercellular junctions [65]. Phosphorylation or decreased mRNA expression is associated with the disassembly of CDH5 molecules and increased vascular permeability. Genetic variations in CDH5, combined with triggering events such as corticosteroid treatment, could explain a proportion of CSCR occurrences among male patients. Schubert et al. concluded that steroid-induced suppression of *CDH5* expression may be associated with genetic variation in the gene and results in the major feature of this relatively common ocular disease, the leakage of fluid into the subretinal space [65].

## Conclusion

Many potential upstream, molecular, and pathological mechanisms may lead to choroidal hyperpermeability in CSCR. These components may work together to create the graded severity seen in CSCR patients. Vortex vein compression/engorgement may play a role in the choriocapillaris dysfunction and subsequent RPE barrier breakdown, CNV formation, and SRF accumulation. Pro-inflammatory cytokines are linked to CSCR, and activation of mineralocorticoid receptors may cause vasodilation of choroidal vessels, leading to pachychoroid and CSCR progression. Systemic conditions such as hypertension and OSA, changes in local and systemic hemodynamic factors, the use of phosphodiesterase inhibitors, and genetic predisposition may additionally factor into choroidal alterations that lead to CSCR. Although we have made large advances in our understanding of CSCR, there is still much to learn regarding this chorioretinal disease. As CSCR may lead to irreversible blindness for affected individuals, it is of utmost importance to continue studying the various potential etiologies of this disease. As we continue to advance our understanding of the molecular underpinnings of CSCR, we may be able to better stratify individuals at larger risk and evaluate optimized treatment plans in their clinical care.

## Data Availability

Not applicable.

## Abbreviations

CSCR: Central serous chorioretinopathy
OCT: Optical coherence tomography
OSA: Obstructive sleep apnea
PNV: Pachychoroid neovasculoapthy
PED: Pigment epithelial detachment
PCV: Polypoidal choroidal vasculopathy
RPE: Retinal pigment epithelium
SRF: Subretinal fluid
